# Dr. Wallace A. Sibley

**Published:** 1900-12

**Authors:** 


Dr. Wallace A. Sibley, of Rochester, N.Y., a well-known physician, died at his home, No. 57 Lake Avenue, October 28, 1900, at the age of 54 years. Organic heart disease, with which he had been afflicted for years, was the attributed cause of his death.

